# The effects of plant-based diets on the body and the brain: a systematic review

**DOI:** 10.1038/s41398-019-0552-0

**Published:** 2019-09-12

**Authors:** Evelyn Medawar, Sebastian Huhn, Arno Villringer, A. Veronica Witte

**Affiliations:** 1 Department of Neurology, Max Planck Institute for Human Cognitive and Brain Sciences, Leipzig, Germany; 2 Berlin School of Mind and Brain, Humboldt-Universität zu Berlin, Berlin, Germany; 3 Charité—Universitätsmedizin Berlin, Humboldt-Universität zu Berlin, Berlin, Germany; 4 Helmholtz Centre for Environmental Research GmbH—UFZ, Leipzig, Germany

**Keywords:** Molecular neuroscience, Psychiatric disorders, Genomics, Human behaviour

## Abstract

Western societies notice an increasing interest in plant-based eating patterns such as vegetarian and vegan, yet potential effects on the body and brain are a matter of debate. Therefore, we systematically reviewed existing human interventional studies on putative effects of a plant-based diet on the metabolism and cognition, and what is known about the underlying mechanisms. Using the search terms “plant-based OR vegan OR vegetarian AND diet AND intervention” in PubMed filtered for clinical trials in humans retrieved 205 studies out of which 27, plus an additional search extending the selection to another five studies, were eligible for inclusion based on three independent ratings. We found robust evidence for short- to moderate-term beneficial effects of plant-based diets versus conventional diets (duration ≤ 24 months) on weight status, energy metabolism and systemic inflammation in healthy participants, obese and type-2 diabetes patients. Initial experimental studies proposed novel microbiome-related pathways, by which plant-based diets modulate the gut microbiome towards a favorable diversity of bacteria species, yet a functional “bottom up” signaling of plant-based diet-induced microbial changes remains highly speculative. In addition, little is known, based on interventional studies about cognitive effects linked to plant-based diets. Thus, a causal impact of plant-based diets on cognitive functions, mental and neurological health and respective underlying mechanisms has yet to be demonstrated. In sum, the increasing interest for plant-based diets raises the opportunity for developing novel preventive and therapeutic strategies against obesity, eating disorders and related comorbidities. Still, putative effects of plant-based diets on brain health and cognitive functions as well as the underlying mechanisms remain largely unexplored and new studies need to address these questions.

## Introduction

### Background

**Fig. 1:**
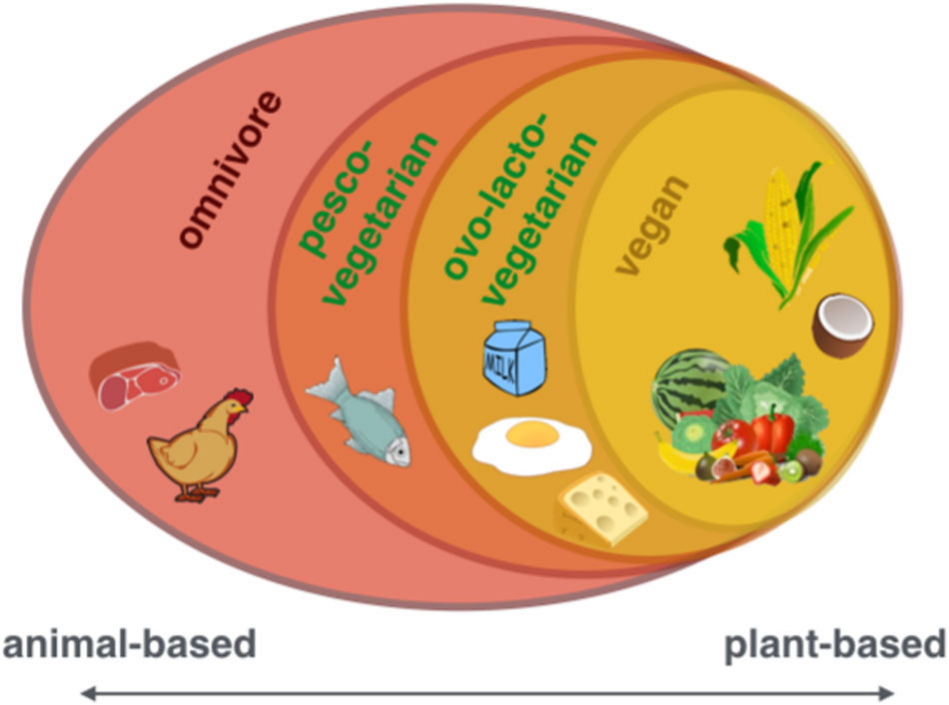
The spectrum of diets including all or only certain types of animal-based products. From left to right: including all food items (omnivore), including all except for meat (pesco-vegetarian) or meat and fish (ovo-lacto-vegetarian) to including only plant-based items (vegan)

**Fig. 2:**
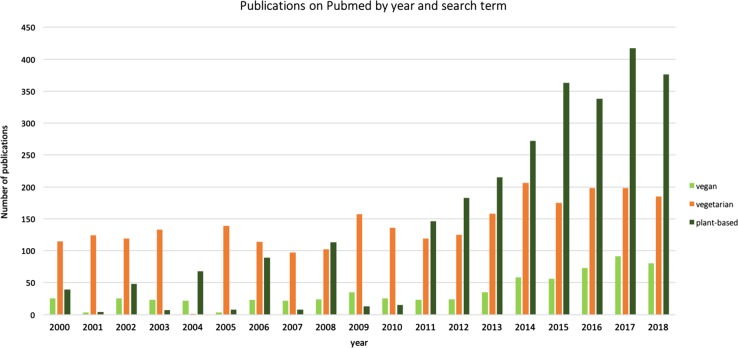
Frequency of publications on PubMed including the search terms “vegan” (in light green), vegetarian (in orange) and plant-based (dark green)—accessed on 19 April 2019

**Fig. 3:**
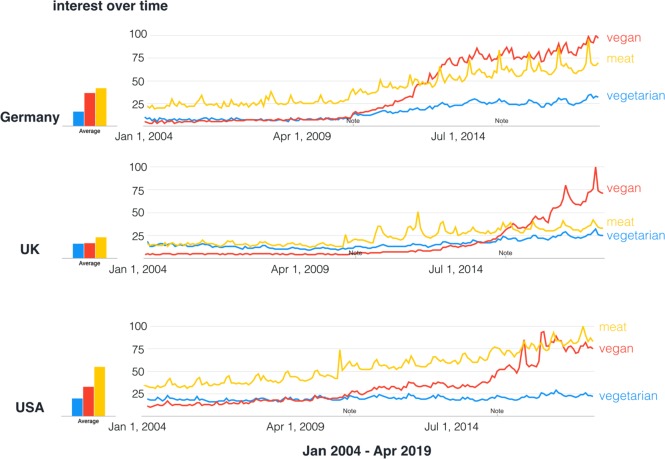
Google Trends Search for search term hits for “vegan”, “vegetarian” and “meat” in Germany (adapted to “vegetarisch”, “vegan” and “fleisch”), the USA and the UK from 2004 to present. Note indicates technical improvements implemented by Google Trends. Data source: Google Trends. Search performed on 18 April 2019

Western societies notice an increasing interest in plant-based eating patterns such as avoiding meat or fish or fully excluding animal products (vegetarian or vegan, see Fig. 1). In 2015, around 0.4−3.4% US adults, 1−2% British adults, and 5−10% of German adults were reported to eat largely plant-based diets^1–4^, due to various reasons (reviewed in ref. ^5^). Likewise, the number of scientific publications on PubMed (Fig. 2) and the public popularity as depicted by Google Trends (Fig. 3) underscore the increased interest in plant-based diets. This increasing awareness calls for a better scientific understanding of how plant-based diets affect human health, in particular with regard to potentially relevant effects on mental health and cognitive functions.

### Study aims

A potential effect of plant-based diets on mortality rate remains controversial: large epidemiological studies like the Adventist studies (*n* = 22,000−96,000) show a link between plant-based diets, lower all-cause mortality and cardiovascular diseases^6,7^, while other studies like the EPIC-Oxford study and the “45 and Up Study” (*n* = 64,000−267,000) show none^8,9^. Yet, many, but not all, epidemiological and interventional human studies in the last decades have suggested that plant-based diets exert beneficial health effects with regard to obesity-related metabolic dysfunction, type 2 diabetes mellitus (T2DM) and chronic low-grade inflammation (e.g. refs. ^6,7,10,11^, for reviews, see refs. ^12–18^). However, while a putative link between such metabolic alterations and brain health through pathways which might include diet-related neurotransmitter precursors, inflammatory pathways and the gut microbiome^19^ becomes increasingly recognized, the notion that plant-based diets exert influence on mental health and cognitive functions appears less documented and controversial^20–24^. We therefore systematically reviewed the current evidence based on available controlled interventional trials, regarded as the gold standard to assess causality, on potential effects of plant-based diets on (a) metabolic factors including the microbiome and (b) neurological or psychiatric health and brain functions. In addition, we aimed to evaluate potential underlying mechanisms and related implications for cognition.

## Methods

We performed a systematic PubMed search with the following search terms “plant-based OR vegan OR vegetarian AND diet AND intervention” with the filter “clinical trial” and “humans”, preregistered at PROSPERO (CRD42018111856; https://www.crd.york.ac.uk/PROSPERO/display_record.php?RecordID=111856) (Suppl. Fig. 1). PubMed was used as search engine because it was esteemed to yield the majority of relevant human clinical trials from a medical perspective. Exclusion criteria were insufficient design quality (such as lack of a control group), interventions without a plant-based or vegetarian or vegan diet condition, intervention with multiple factors (such as exercise and diet), and the exclusive report of main outcomes of no interest, such as dietary compliance, nutrient intake (such as vitamins or fiber intake), or nonmetabolic (i.e., not concerning glucose metabolism, lipid profile, gastrointestinal hormones or inflammatory markers) or non-neurological/psychiatric disease outcomes (e.g. cancer, caries).

Studies were independently rated for eligibility into the systematic review by three authors based on reading the abstract and, if needed, methods or other parts of the publication. If opinions differed, a consensus was reached through discussion of the individual study. This yielded 27 eligible out of 205 publications; see Table 1 for details. To increase the search radius for studies dealing with microbial and neurological/psychiatric outcomes, we deleted the search term “intervention”, which increased the number of studies by around one third, and checked for studies with “microbiome/microbiota”, “mental”, “cognitive/cognition” or “psychological/psychology” in the resulting records. Through this, we retrieved another five studies included in Table 1. Further related studies were reviewed based on additional nonsystematic literature search.

**Table 1:** Intervention studies on the effect of plant-based diets

Author Year	Study design	*n* patients	*n* healthy	Nature of intervention, and if calorie-restricted	Duration of intervention	Measures	Effect of intervention	Favoring vegan diet
Weight loss, blood-based metabolic markers								
Turner-McGrievy et al. (2007)^139^	RCT; overweight postmenopausal women: low-fat vegan vs. National Cholesterol Education Program diet two replications		62; first run 28 (14 vs. 14), second run 34 (17 vs. 17)	Low-fat vegan diet (unrestricted): − fruits, vegetables, legumes, grains − animal products proscribed − limit high-fat plant foods vs. National Cholesterol Education Program diet (unrestricted): − see guidelines	14 weeks (24 months follow-up)	Body weight	− weight loss higher in vegan group at year 1 and year 2	+
Burke et al. (2008)^140^	RCT; obese subjects; four groups: freely chosen vegetarian vs. freely chosen conventional vs. assigned vegetarian vs. assigned conventional		178 (48 vs. 35 vs. 48 vs. 45)	Vegetarian (restricted): − no meat, poultry, fish vs. Standard behavioral therapy, group sessions led by dietician/physiologist/nurse/behavioral scientist − monitoring of physical activity and calorie/fat content of foods − cooking magazines provided	18 months	Body weight	− weight loss higher in both groups that were assigned to a certain diet − trend to higher weight loss in both vegetarian groups − all groups showed significant weight loss	+
Barnard et al. (2009)^141^	RCT; T2DM patients; two groups: Vegan vs. conventional restrictive diet	99 (49 vs. 50)		Vegan (unrestricted): − 10% fat, 15% protein, 75% carbohydrates − daily cholesterol intake < 50 mg − vegetables, fruit, grains, legumes − no animal products, fatty foods and high-glycaemic index foods vs. Conventional: −<7% fat, 15−20% protein, 60−70% carbohydrates − meal plan with dietician, 3-day dietary record	74 weeks	Body weight, blood measures	− significant weight loss in both groups (trend towards stronger effect in vegan group) − lower HbA1C, total-/LDL-/ and non-HDL-cholesterol after intervention in both groups, trend towards lower HbA1C in vegan group − controlling for medication changes led to significantly greater reductions in HbA1C, total- and LDL-cholesterol in vegan group	+
Elkan et al. (2008)^40^	Rheumatoid arthritis patients	66 (38 vs. 28)		Gluten-free vegan diet (protein energy level was 10% of the total energy intake, the carbohydrates 60%, and fat 30%; contained vegetables, root vegetables, nuts, fruits) vs. well-balanced non-vegan (contained 10 to 15% protein, 55 to 60% carbohydrate, no more than 30% fat)	12 months	Body weight, blood measures	− lower BMI, LDL, TC and higher anti-PC IgM in the vegan diet group	+
Marniemi et al. (1990)^142^	Moderately obese subjects		110 in total (31 vs. 37 vs. 42)	Lactoovo (1200 kcal/day) vs. mixed diet (1200 kcal/day) vs. control (no intervention)	12 months	Body weight, blood measures	− Weight-reduction, improved lipid metabolism in both intervention groups, stronger effects in mixed diet compared to lactovegetarian diet	−
Acharya et al. (2013)^143^	Pilot study for RCT; overweight and obese subject		143 in total (79 vs. 64)	Standard calorie- and fat-restricted diet vs. calorie- and fat-restricted lacto-ovo-vegetarian diet	6 months	Body weight	− no significant effect on weight dependent on diet	o
Wright et al. (2017)^144^	RCT; mid-age to old T2DM and overweight patients; whole food plant-based unrestricted vs. usual care	65 (32 vs. 33)		Low-fat plant-based: − 7−15% fat − whole grains, legumes, vegetables, fruits − calorie-unrestricted − avoid animal products and refined oils, high-fat plant foods, sugar, salt, caffeine − 50 μg/day vitamin B12	6 months	Body weight, blood measures	− reduced BMI and mean cholesterol in plant-based group	+
Jenkins et al. (2014)^29^	RCT; overweight hyperlipidemic patients; low-carb vegan vs. high-carb lacto-ovo	39 (19 vs. 20)		− caloric restriction to 60% of estimated caloric requirements low-carb vegan: − 26% carbohydrates, 31% plant protein, 43% fat vs. high-carb lacto-ovo-vegetarian: − 58% carbohydrates, 16% protein, 25% fat	6 months	Body weight, blood measures	− higher weight loss and lower LDL and TG for low-carb vegan group after 1 month^31^: − weight loss reduced in both groups (about 4.0 kg) (n.s. difference across groups) − more reduced LDL, TC, apolipoproteins for plant-based group	+
Turner-McGrievy et al. (2015)^33,161^	RCT; healthy overweight subjects 25-49.9 kg/m^2^; calorie-unrestricted		50 (12 vs. 13 vs. 13 vs. 12)	− avoid fast foods and processed foods; self-based diets − all groups received weekly dietary sessions except for the omnivore group (kept following their usual diet) vegan: − no animal products, focus on plant-based foods vs. vegetarian: − no meat, fish, poultry, but eggs and dairy vs. pesco-vegetarian: − no meat, poultry, but fish, shellfish, eggs, dairy vs. semi-vegetarian: − all foods, red meat limited to 1/week and poultry limited to <5/week	6 months	Body weight, blood measures	− higher weight loss in vegan group (particularly decreased fat and saturated fat)	+
Turner-McGrievy et al. (2014)^145^	RCT; overweight subjects with polycystic syndrome: vegan vs. low-calorie diet	18 (9 vs. 9)		Vegan: − exclude all animal products, limit high glycaemic-index foods vs. Low-calorie: − restricted to 1200−1500 kcal/day depending on body weight − assessed by weekly 24 h recall	6 months	Body weight, polycystic syndrome	− higher weight loss at 3 months for vegan group (not after 6 months) − lower energy intake after 6 months for vegan group (lower fat, lower protein) − no changes for polycystic syndrome	+/o
Kahleova et al. (2011)^146^	RCT; T2DM patients; two groups: vegetarian vs. conventional diabetic diet	74 (37 vs. 37)		Vegetarian (restricted) vs. Conventional (restricted) − all meals provided − after 12 weeks physical exercise added	6 months	Body weight, polycystic syndrome	− reduced medication, higher weight loss, increased insulin sensitivity, reduced visceral and subcutaneous fat, increase in plasma adiponectin, decrease in leptin in the vegan group	+
Ferdowsian et al. (2010)^147^	RCT; overweight and/or T2DM patients: low-fat vegan diet vs. control; onsite	113		Low-fat vegan: − no meat, poultry, fish, dairy, eggs, <5% saturated fat, <25% total fat, < 50 mg cholesterol daily − multivitamin supplement (incl. B12) vs. control: − usual diet	5,5 months	Body weight	− reduced body weight and waist circumference in intervention group	+
Mishra et al. (2013) (same sample as Agarwal et al. (2015) and partly overlapping with Ferdowsian et al. (2010))^147–149^	RCT; overweight and/or T2DM patients; multicomponent worksite intervention; low-fat vegan vs. usual diet	291 at 4 sites; (142 vs. 149)		low-fat vegan (unrestricted): − avoid all animal products, minimize added oils, favor whole grains − vitamin B12 and multivitamin supplements vs. Control: − usual diet; no instruction	18 weeks	Blood measures	− lower total cholesterol in vegan group	+
Kahleova et al. (2018)^150^	RCT; T2DM patients	74 (37 vs. 37)		vegetarian diet (−500 kcal/day) vs. control isocaloric conventional anti-diabetic diet (−500 kcal/day)	16 weeks	Anthropo-metric measures	− greater reduction in total leg area for thigh adipose tissue distribution after vegetarian diet	+
Lee et al. (2016)^28^	RCT; healthy Korean subjects; two groups: Vegan vs. conventional restrictive diet		106 (46 vs. 47)	Vegan (unrestricted): (1) ingest unpolished rice (brown rice); (2) avoid polished rice (white rice); (3) avoid processed food made of rice flour or wheat flour; (4) avoid all animal food products (i.e., meat, poultry, fish, daily goods, and eggs); and (5) favor low-glycemic index foods (e.g., legumes, legumes-based foods, green vegetables, and seaweed) vs. Conventional (restricted) (1) restrict their individualized daily energy intake based on body weight, physical activity, need for weight control, and compliance; (2) total calorie intake comprised 50–60% carbohydrate, 15–20% protein (if renal function is normal), <25% fat, <7% saturated fat, minimal trans-fat intake, and ≤200 mg/day cholesterol	12 weeks	Body weight, blood measures	− significantly larger reduction of HbA1C levels, trends towards lower BMI and lower waist circumference in the vegan intervention group	+
Barnard et al. (2000)^151^	RCT; premenopausal women	51 (35)		low-fat vegetarian (10% fat) vs. normal diet incl. a placebo pill	3 months	Blood measures	− decreased LDL, HDL, TC after 10% fat-vegetarian diet	+
Rauma et al.^39^	Rheumatoid arthritis patients	43 (22 vs. 21)		vegan vs. control (usual diet)	3 months	Body weight, urine measures	− 9% reduction of body weight in the vegan group	+
Gardner et al. (2005)^152^	RCT; hypercholesterolemic outpatients 30−65 years	120 (59 vs. 61)		low-fat diet (incl. animal products) vs. low-fat plus diet (more veggie, legumes, whole grains)	1 month	Blood measures	− lower TC, LDL for low-fat plus (plant-based) diet	+
Macknin et al. (2015)^153^	Randomized; obese hypercholesterolemic children and their parents	30 (16 vs. 14)		plant-based no added fat diet (PB) vs. American Heart Association Diet (AHA)	1 month	Body weight, blood measures	− lower BMI and hsCRP levels as well as higher waist circumference in the plant-based and no-added fat diet condition in children, − lower cholesterol, LDL and HbA1c in the plant-based and no-added fat diet condition in parents	+/o
Sciarrone et al. (1993)^154^	Parallel randomized trial, healthy men		20 (10 vs. 10)	lacto-ovo-vegetarian diet vs. omnivorous diet − initial 2 weeks under caloric restriction, afterwards unrestricted	6 weeks	Body weight, blood measures	− no significant differences in body weight, glucose, insulin or catecholamines between groups	o
Alleman et al. (2013)^155^	Interventional study, healthy subjects		29 (16 vs. 13)	traditional (vegan) vs. modified Daniel Fast diet (incl. daily meat and dairy)	3 weeks	Body weight, blood measures	− no significant weight changes after dietary intervention for neither condition − both diets show improvement of blood lipids, inflammation markers	o
Neacsu et al. (2014)^156^	Within-subject cross-over design; obese men		20 in total	meat-based high-protein diet vs. vegetarian soy high-protein diet (both diets: 30% protein, 30% fat, 40% carbohydrate)	2 weeks	Body weight, blood measures	− n. s. differences between weight loss and gut hormone profile	o
Koebnick et al. (2004)^157^	RCT; healthy subjects; site-based study		32 in total	low-fat plant-based (20% fat) vs. control	1 week	Blood measures	− reduced TC, LDL, TG in vegan diet	+
Microbiome								
David et al. (2014)^35^	Within-subject cross-over design, healthy, young volunteers		10	exclusively plant-based diet (unrestricted) vs. nearly exclusively animal-based diet (unrestricted)	5 days	16S rRNA gene sequencing (stool samples)	Higher abundance of bile-tolerant microorganisms (Alistipes, Bilophila, Bacteroides) and decreased levels of Firmicutes (Roseburia, Eubacterium rectale, Ruminococcus bromii).	?
Neurological/psychiatric disease outcomes and brain functions								
Karlsson et al. (1994)^41^	RCT; moderately obese women		60	1300 kcal lacto-vegetarian diet vs. 1300 kcal conventional weight-reducing diet	3, 8, 24 months	Psychological measures incl. mental well-being, functional status; body weight	− no significant differences between groups on psychological measures and BMI	o
Kjeldsen-Kragh et al. (1994)^158^	RCT; rheumatoid arthritis patients, vegetarian vs. omnivorous diet	53 (27 vs. 26)		− vegetarian diet (fasting 7−10 days, gluten free vegan diets for 3.5 months, afterwards lacto-vegetarian diet vs. - normal omnivorous diet	13 months	General Health Questionnaire	− improvements in psychological distress including depression and anxiety subscores in the vegetarian group	+
Yadav et al. (2016)^38^	RCT; multiple sclerosis patients	61 (32 vs. 29)		very low-fat plant-based diet: − starchy plant foods, 10% fat, 14% protein, 76% carbohydrates (no meat, fish, eggs, dairy products or vegetable oils) vs. control: − usual diet − assessed by FFQ and meetings with dietician	12 months	Brain MRI, fatigue, body weight, blood sample	− no clear effect on brain MRI outcomes; improvement of fatigue, weight status and metabolic markers in the vegan group	o/+
Bunner et al. (2014)^159^	RCT; cross-over trial migraine patients; Low-fat vegan vs. placebo	42 in total		Vegan diet: Favored intake of whole grains, lentils, certain vegetables; avoidance of all animal products, nuts and seeds, alcohol, coffee vs. Placebo: 10 mcg alpha-linolenic acid and 10 mcg vitamin E/day	9 months	Headache pain measured with The Patient’s Global Impression of Change	− improvement of migraine during last 2 weeks in the vegan group	+
Kahleova et al. (2013)^160^	Randomized, open, parallel design, T2DM patients, vegetarian vs. control group	74 (37 vs. 37)		vegetarian diet (−500 kcal/day) vs. control isocaloric conventional anti-diabetic diet (−500 kcal/day)	24 weeks	Quality of life, depressive symptoms, eating behavior	− improved quality of life, dietary restraint and disinhibition and lower depression scores in the vegetarian group	+
Agarwal et al. (2015)^23^	RCT; overweight and/or T2DM patients; multicomponent worksite intervention; low-fat vegan vs. usual diet	291 at 4 sites; (142 vs. 149)		low-fat vegan (unrestricted): − avoid all animal products, minimize added oils, favor whole grains − vitamin B12 and multivitamin supplements vs. Control: − usual diet; no instruction	18 weeks	Depression, anxiety, fatigue, emotional well-being	− all measures significantly improved in the vegan group	+
Kaartinen et al. (2000)^37^	Non-randomized; fibromyalgia patients	32 (18 vs. 15)		low-salt, raw vegan diet vs. omnivorous diet	3 months	Disease improvement, urine and blood measures	− less pain, improved joint stiffness and quality of sleep, decreased weight, TC, and urine sodium in the vegan diet group	+
Beezhold et al. (2012)^42^	Healthy subjects; omnivorous		39 (in locks at 3, i.e. 13 in each group)	control group consuming meat, fish, and poultry daily (OMN) vs. a group consuming fish 3−4 times weekly but avoiding meat and poultry (FISH) vs. a vegetarian group avoiding meat, fish, and poultry (VEG)	2 weeks	Stress, depression, mood, anxiety, blood levels	− decrease in stress, anxiety and improved mood in vegan group − decreased fatty acids, increased *n*−6 to *n*−3 ratio and decrease in alpha-linoleic acid in the VEG compared to OMN group	+

## Section I: Effects of plant-based diets on body and brain outcomes

### Results based on interventional studies on metabolism, microbiota and brain function

Overall, the vast majority of studies included in this systematic review reported a short-term beneficial effect of plant-based dietary interventions (study duration 3−24 months) on weight status, glucose, insulin and/or plasma lipids and inflammatory markers, whereas studies investigating whether plant-based diets affect microbial or neurological/psychiatric disease status and other brain functions were scarce and rather inconclusive (Table 1).

More specifically, 19 out of 32 studies dealing with T2DM and/or obese subjects and seven out of 32 dealing with healthy subjects observed a more pronounced weight loss and metabolic improvements, such as lowering of glycated hemoglobin (HbA1c)—a long-term marker for glucose levels—decreased serum levels of low-density (LDL) and high-density lipoproteins (HDL) and total cholesterol (TC), after a plant-based diet compared to an omnivore diet. This is largely in line with recent meta-analyses indicating beneficial metabolic changes after a plant-based diet^25–27^.

For example, Lee et al. found a significantly larger reduction of HbA1c and lower waist circumference after vegan compared to conventional dieting^28^. Jenkins et al. found a disease-attenuating effect in hyperlipidemic patients after 6 months adopting a low-carbohydrate plant-based diet compared to a high-carbohydrate lacto-ovo-vegetarian diet^29,30^. However, lower energy intake in the vegan dieters might have contributed to these effects. Yet, while a plant-based diet per se might lead to lower caloric intake, other studies observed nonsignificant trends toward higher effect sizes on metabolic parameters after a vegan diet, even when caloric intake was comparable: two studies in T2DM patients^31,32^ compared calorie-unrestricted vegan or vegetarian to calorie-restricted conventional diets over periods of 6 months and 1.5 years, respectively, in moderate sample sizes (*n* ~ 75−99) with similar caloric intake achieved in both diet groups. Both studies indicated stronger effects of plant-based diets on disease status, such as reduced medication, improved weight status and increased glucose/insulin sensitivity, proposing a diabetes-preventive potential of plant-based diets. Further, a five-arm study comparing four types of plant-based diets (vegan, vegetarian, pesco-vegetarian, semi-vegetarian) to an omnivore diet (total *n* = 63) in obese participants found the most pronounced effect on weight loss for a vegan diet (−7.5 ± 4.5% of total body weight)^33^. Here, inflammation markers conceptualized as the dietary inflammatory index were also found to be lower in vegan, vegetarian and pesco-vegetarian compared to semi-vegetarian overweight to obese dieters^33^.

Intriguingly, these results^28–33^ cohesively suggest that although caloric intake was similar across groups, participants who had followed a vegan diet showed higher weight loss and improved metabolic status.

As a limitation, all of the reviewed intervention studies were carried out in moderate sample sizes and over a period of less than 2 years, disregarding that long-term success of dietary interventions stabilizes after 2−5 years only^34^. Future studies with larger sample sizes and tight control of dietary intake need to confirm these results.

Through our systematic review we retrieved only one study that added the gut microbiome as novel outcome for clinical trials investigating the effects of animal-based diets compared to plant-based diets. While the sample size was relatively low (*n* = 10, cross-over within subject design), it showed that changing animal- to plant based diet changed gut microbial activity towards a trade-off between carbohydrate and protein fermentation processes within only 5 days^35^. This is in line with another controlled-feeding study where microbial composition changes already occurred 24 h after changing diet (not exclusively plant-based)^36^. However, future studies incorporating larger sample sizes and a uniform analysis approach of microbial features need to further confirm the hypothesis that a plant-based diet ameliorates microbial diversity and health-related bacteria species.

Considering neurological or psychiatric diseases and brain functions, the systematic review yielded in six clinical trials of diverse clinical groups, i.e. migraine, multiple sclerosis, fibromyalgia and rheumatoid arthritis. Here, mild to moderate improvement, e.g. measured by antibody levels, symptom improvement or pain frequency, was reported in five out of six studies, sometimes accompanied by weight loss^37–40^ (Table 1). However, given the pilot character of these studies, indicated by small sample sizes (*n* = 32−66), lack of randomization^37^, or that the plant-based diet was additionally free of gluten^40^, the evidence is largely anecdotal. One study in moderately obese women showed no effects on psychological outcomes^41^, two studies with obese and nonobese healthy adults indicated improvements in anxiety, stress and depressive symptom scores^23,24^. Taken together, the current evidence based on interventional trials regarding improvements of cognitive and emotional markers and in disease treatment for central nervous system disorders such as multiple sclerosis or fibromyalgia remains considerably fragmentary for plant-based diets.

Among observational studies, a recent large cross-sectional study showed a higher occurrence of depressive symptoms for vegetarian dieters compared to nonvegetarians^20^. Conversely, another observational study with a sample of about 80% women found a beneficial association between a vegan diet and mood disturbance^24^.

Overall, the relationship between mental health (i.e. depression) and restrictive eating patterns has been the focus of recent research^20–22,24,42^; however, causal relationships remain uninvestigated due to the observational design.

### Underlying mechanisms linking macronutrient intake to metabolic processes

**Fig. 4:**
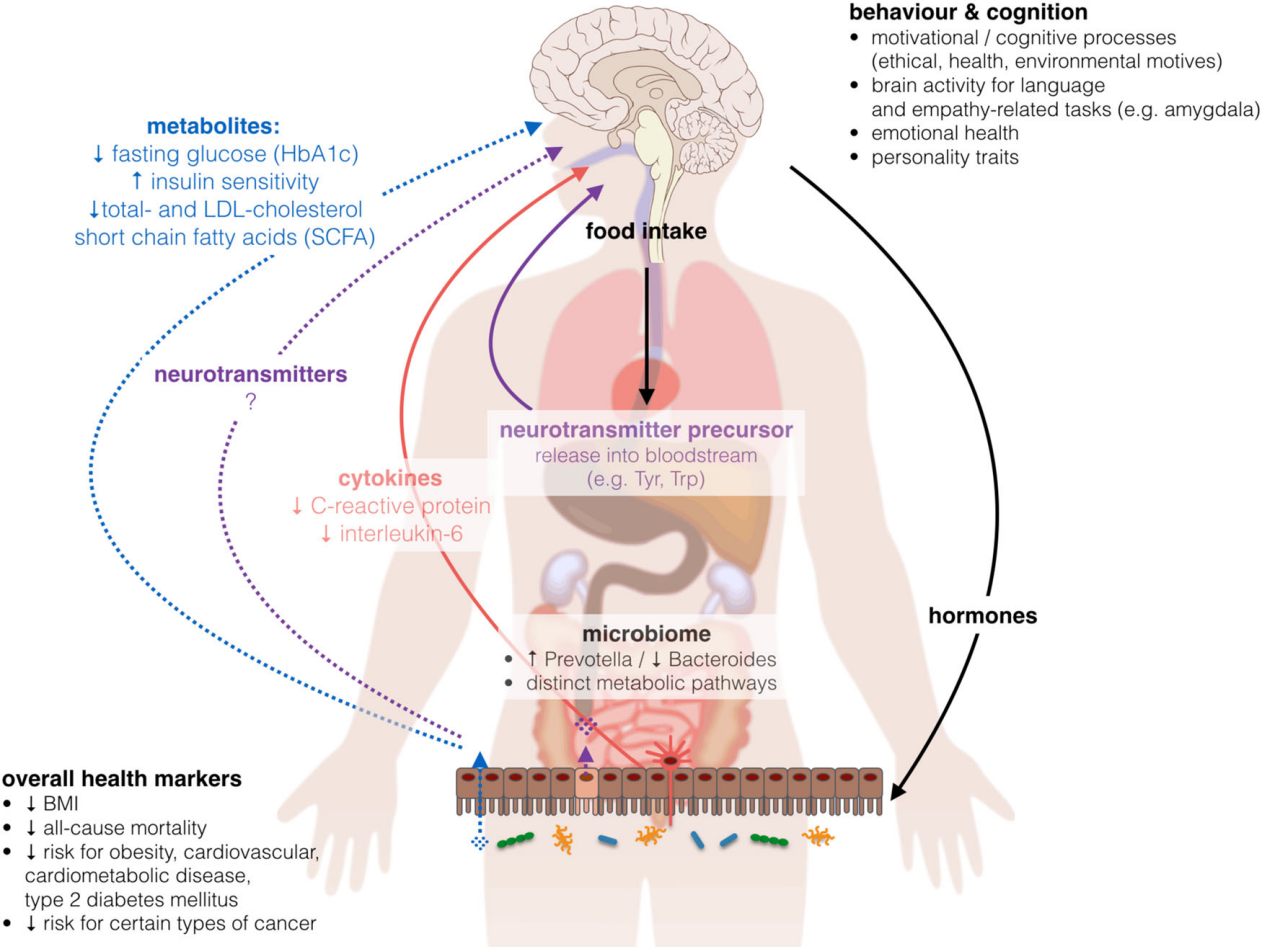
The effects of a plant-based diet on the microbiome−gut−brain axis including the here reviewed effects on overall health, microbial composition and activity, behavior and cognition. BMI body-mass-index, HbA1c hemoglobin A1c, LDL-cholesterol low-density lipoprotein cholesterol, Trp tryptophan, Tyr tyrosine. Images from commons.wikimedia.org, “Brain human sagittal section” by Lynch 2006 and “Complete GI tract” by Häggström 2008, “Anatomy Figure Vector Clipart” by http://moziru.com

On the one hand, nutrient sources as well as their intake ratios considerably differ between plant-based and omnivore diets (Suppl. Table 1), and on the other hand, dietary micro- and macromolecules as well as their metabolic substrates affect a diversity of physiological functions, pointing to complex interdependencies. Thus, it seems difficult to nail down the proposed beneficial effects of a plant-based diet on metabolic status to one specific component or characteristic, and it seems unlikely that the usually low amount of calories in plant-based diets could explain all observed effects. Rather, plant-based diets might act through multiple pathways, including better glycemic control^43^, lower inflammatory activity^44^ and altered neurotransmitter metabolism via dietary intake^45^ or intestinal activity^46^ (Fig. 4).

On the macronutrient level, plant-based diets feature different types of fatty acids (mono- and poly-unsaturated versus saturated and trans) and sugars (complex and unrefined versus simple and refined), which might both be important players for mediating beneficial health effects^18^. On the micronutrient level, the EPIC-Oxford study provided the largest sample of vegan dieters worldwide (*n*(vegan) = 2396, *n*(total) = 65,429) and showed on the one hand lower intake of saturated fatty acids (SFA), retinol, vitamin B12 and D, calcium, zinc and protein, and on the other hand higher intake of fiber, magnesium, iron, folic acid, vitamin B1, C and E in vegan compared to omnivore dieters^47^. Other studies confirmed the variance of nutrient intake across dietary groups, i.e. omnivores, vegetarians and vegans, showing the occurrence of critical nutrients for each group^48,49^. Not only the amount of SFA but also its source and profile might be important factors regulating metabolic control (reviewed in ref. ^14^), for example through contributing to systemic hyperlipidemia and subsequent cardiovascular risk. Recently, it has been shown in a 4-week intervention trial that short-term dietary changes favoring a diet high in animal-based protein may lead to an increased risk for cardiovascular derangements mediated by higher levels of trimethylamine N-oxide (TMAO), which is a metabolite of gut bacteria-driven metabolic pathways^50^.

Secondly, high fiber intake from legumes, grains, vegetables and fruits is a prominent feature of plant-based diets (Table 1), which could induce beneficial metabolic processes like upregulated carbohydrate fermentation and downregulated protein fermentation^35^, improved gut hormonal-driven appetite regulation^51–55^, and might prevent chronic diseases such as obesity and T2DM by slowing down digestion and improving lipid control^56^. A comprehensive review including evidence from 185 prospective studies and 58 clinical trials concluded that risk reduction for a myriad of diseases (incl. CVD, T2DM, stroke incidence) was greatest for daily fiber intake between 25 and 29 g^57^. Precise evidence for underlying mechanisms is missing; however, more recently it has been suggested that high fiber intake induces changes on the microbial level leading to lower long-term weight gain^58^, a mechanism discussed below.

The reason for lower systemic inflammation in plant-based dieters could be due to the abundance of antiinflammatory molecule intake and/or avoidance of proinflammatory animal-derived molecules. Assessing systemic inflammation is particularly relevant for medical conditions such as obesity, where it has been proposed to increase the risk for cardiovascular disease^59,60^. In addition, higher C-reactive protein (CRP) and interleukin-6 (IL-6) levels have been linked with measures of brain microstructure, such as microstructural integrity and white matter lesions^61–63^ and higher risk of dementia^64^, and recent studies point out that a diet-related low inflammatory index might also directly affect healthy brain ageing^65,66^.

Interventional studies that focus on plant- versus meat-based proteins or micronutrients and potential effects on the body and brain are lacking. A meta-analysis including seven RCTs and one cross-sectional studies on physical performance and dietary habits concluded that a vegetarian diet did not adversely influence physical performance compared to an omnivore diet^67^. An epidemiological study by Song et al.^11^ estimated that statistically replacing 3% of animal protein, especially from red meat or eggs, with plant protein would significantly improve mortality rates. This beneficial effect might however not be explained by the protein source itself, but possibly by detrimental components found in meat (e.g. heme-iron or nitrosamines, antibiotics, see below).

Some studies further hypothesized that health benefits observed in a plant-based diet stem from higher levels of fruits and vegetables providing phytochemicals or vitamin C that might boost immune function and eventually prevent certain types of cancer^68–70^. A meta-analysis on the effect of phytochemical intake concluded a beneficial effect on CVD, cancer, overweight, body composition, glucose tolerance, digestion and mental health^71^. Looking further on the impact of micronutrients and single dietary compounds, there is room for speculation that molecules, that are commonly avoided in plant-based diets, might affect metabolic status and overall health, such as opioid-peptides derived from casein^72^, pre- and probiotics^73,74^, carry-over antibiotics found in animal products^75,76^ or food-related carcinogenic toxins, such as dioxin found in eggs or nitrosamines found in red and processed meat^77,78^. Although conclusive evidence is missing, these findings propose indirect beneficial effects on health deriving from plant-based compared to animal-based foods, with a potential role for nonprotein substances in mediating those effects^18^. While data regarding chemical contaminant levels (such as crop pesticides, herbicides or heavy metals) in different food items are fragmentary only, certain potentially harmful compounds may be more (or less) frequently consumed in plant-based diets compared to more animal-based diets^79^. Whether these differences lead to systematic health effects need to be explored.

Taken together, the reviewed studies indicating effects of plant-based diets through macro- and micronutrient intake reveal both the potential of single ingredients or food groups (low SFA, high fiber) and the immense complexity of diet-related mechanisms for metabolic health. As proposed by several authors, benefits on health related to diet can probably not be viewed in isolation for the intake (or nonintake) of specific foods, but rather by additive or even synergistic effects between them (reviewed in refs. ^12,80^). Even if it remains a challenging task to design long-term RCTs that control macro- and micronutrient levels across dietary intervention groups, technological advancements such as more fine-tuned diagnostic measurements and automated self-monitoring tools, e.g. automatic food recognition systems^81^ and urine-related measures of dietary intake^82^, could help to push the field forward.

### Nutrients of particular interest in plant-based diets

As described above, plant-based diets have been shown to convey nutritional benefits^48,49^, in particular increased fiber, beta carotene, vitamin K and C, folate, magnesium, and potassium intake and an improved dietary health index^83^. However, a major criticism of plant-based diets is the risk of nutrient deficiencies for specific micronutrients, especially vitamin B12, a mainly animal-derived nutrient, which is missing entirely in vegan diets unless supplemented or provided in B12-fortified products, and which seems detrimental for neurological and cognitive health when intake is low. In the EPIC-Oxford study about 50% of the vegan dieters showed serum levels indicating vitamin B12 deficiency^84^. Along other risk factors such as age^85^, diet, and plant-based diets in particular, seem to be the main risk factor for vitamin B12 deficiency (reviewed in ref. ^86^), and therefore supplementing vitamin B12 for these risk groups is highly recommended^87^. Vitamin B12 is a crucial component involved in early brain development, in maintaining normal central nervous system function^88^ and suggested to be neuroprotective, particularly for memory performance and hippocampal microstructure^89^. One hypothesis is that high levels of homocysteine, that is associated with vitamin B12 deficiency, might be harmful to the body. Vitamin B12 is the essential cofactor required for the conversion of homocysteine into nonharmful components and serves as a cofactor in different enzymatic reactions. A person suffering from vitamin B12 insufficiency accumulates homocysteine, lastly promoting the formation of plaques in arteries and thereby increasing atherothrombotic risk^90^, possibly facilitating symptoms in patients of Alzheimer’s disease^91^. A meta-analysis found that vitamin B12 deficiency was associated with stroke, Alzheimer’s disease, vascular dementia, Parkinson’s disease and in even lower concentrations with cognitive impairment^92^, supporting the claim of its high potential for disease prevention when avoided or treated^93^. Further investigations and longitudinal studies are needed, possibly measuring holotranscobalamin (the active form of vitamin B12) as a more specific and sensitive marker for vitamin B12 status^94^, to examine in how far nonsupplementing vegan dieters could be at risk for cardiovascular and cognitive impairment.

Similar health dangers can stem from iron deficiency, another commonly assumed risk for plant-based dieters and other risk groups such as young women. A meta-analysis on 24 studies proposes that although serum ferritin levels were lower in vegetarians on average, it is recommended to sustain an optimal ferritin level (neither too low nor too high), calling for well-monitored supplementation strategies^95^. Iron deficiency is not only dependent on iron intake as such but also on complimentary dietary factors influencing its bioavailability (discussed in ref. ^95^). The picture remains complex: on the one hand iron deficiency may lead to detrimental health effects, such as impairments in early brain development and cognitive functions in adults and in children carried by iron-deficient mothers^96^ and a possible role for iron overload in the brain on cognitive impairment on the other hand^97^. One study showed that attention, memory and learning were impaired in iron-deficient compared to iron-sufficient women, which could be restored after a 4-month oral iron supplementation (*n* = 118)^98^. Iron deficiency-related impairments could be attributed to anemia as an underlying cause, possibly leading to fatigue, or an undersupply of blood to the brain or alterations in neurobiological and neuronal systems^99^ provoking impaired cognitive functioning.

This leads to the general recommendation to monitor health status by frequent blood tests, to consult a dietician to live healthily on a plant-based diet and to consider supplements to avoid nutrient deficiencies or nutrient-overdose-related toxicity. All in all, organizations such as the Academy of Nutrition and Dietetics^100^ and the German Nutrition Society do not judge iron as a major risk factor for plant-based dieters^101^.

## Section II: Effects of diet on the gut microbiome

### The link between diet and microbial diversity

Another putative mechanistic pathway of how plant-based diets can affect health may involve the gut microbiome which has increasingly received scientific and popular interest, lastly not only through initiatives such as the Human Microbiome Project^102^. A common measure for characterizing the gut community is enterotyping, which is a way to stratify individuals according to their gut bacterial diversity, by calculating the ratio between bacterial genera, such as Prevotella and Bacteroides^103^. While interventional controlled trials are still scarce, this ratio has been shown to be conclusive for differentiating plant-based from animal-based microbial profiles^36^. Specifically, in a sample of 98 individuals, Wu et al.^36^ found that a diet high in protein and animal fats was related to more Bacteroides, whereas a diet high in carbohydrates, representing a plant-based one, was associated with more Prevotella. Moreover, the authors showed that a change in diet to high-fat/low-fiber or to low-fat/high-fiber in ten individuals elicited a change in gut microbial enterotype with a time delay of 24 h only and remained stable over 10 days, however not being able to switch completely to another enterotype^36^. Another strictly controlled 30-day cross-over interventional study showed that a change in diet to either an exclusively animal-based or plant-based diet promoted gut microbiota diversity and genetic expression to change within 5 days^35^. Particularly, in response to adopting an animal-based diet, microbial diversity increased rapidly, even overshadowing individual microbial gene expression. Beyond large shifts in overall diet, already modest dietary modifications such as the daily consumption of 43 g of walnuts, were able to promote probiotic- and butyric acid-producing bacterial species in two RCTs, after 3 and 8 weeks respectively^104,105^, highlighting the high adaptability of the gut microbiome to dietary components. The Prevotella to Bacteroides ratio (P/B) has been shown to be involved in the success of dietary interventions targeting weight loss, with larger weight loss in high P/B compared to low P/B in a 6-month whole-grain diet compared to a conventional diet^106^. Only recently, other microbial communities, such as the salivary microbiome, have been shown to be different between omnivores and vegan dieters^107^, opening new avenues for research on adaptable mechanisms related to dietary intake.

### A continuum in microbial diversity dependent on diet

Plant-based diets are supposed to be linked to a specific microbial profile, with a vegan profile being most different from an omnivore, but not always different from a vegetarian profile (reviewed in ref. ^15^). Some specifically vegan gut microbial characteristics have also been found in a small sample of six obese subjects after 1 month following a vegetarian diet, namely less pathobionts, more protective bacterial species improving lipid metabolism and a reduced level of intestinal inflammation^108^. Investigating long-term dietary patterns a study found a dose-dependent effect for altered gut microbiota in vegetarians and vegans compared to omnivores depending on the quantity of animal products^109^. The authors showed that gut microbial profiles of plant-based diets feature the same total number but lower counts of Bacteroides, Bifidobacterium, *E. coli* and Enterobacteriaceae compared to omnivores, with the biggest difference to vegans. Still today it remains unclear, what this shift in bacterial composition means in functional terms, prompting the field to develop more functional analyses.

In a 30-day intervention study, David et al. found that fermentation processes linked to fat and carbohydrate decomposition were related to the abundance of certain microbial species^35^. They found a strong correlation between fiber intake and Prevotella abundance in the microbial gut. More recently, Prevotella has been associated with plant-based diets^110^ that are comparable to low-fat/high-fiber diets^111^ and might be linked to the increased synthesis of short-chain fatty acids (SCFA)^112^. SCFAs are discussed as putative signaling molecules between the gut microbiome and the receptors, i.e. free fatty acid receptor 2 (FFA2)^51^, found in host cells across different tissues^113^ and could therefore be one potential mechanism of microbiome−host communication.

The underlying mechanisms of nutrient decomposition by Prevotella and whether abundant Prevotella populations in the gut are beneficial for overall health remain unknown. Yet it seems possible that an increased fiber intake and therefore higher Prevotella abundance such as associated with plant-based diets is beneficial for regulating glycemic control and keeping inflammatory processes within normal levels, possibly due to reduced appetite and lower energy intake mediated by a higher fiber content^114^. Moreover, it has been brought forward that the microbiome might influence bodily homeostatic control, suggesting a role for the gut microbiota in whole-body control mechanisms on the systemic level. Novel strategies aim to develop gut-microbiota-based therapies to improve bodily states, e.g. glycemic control^115^, based on inducing microbial changes and thereby eliciting higher-level changes in homeostasis. While highly speculative, such strategies could in theory also exert changes on the brain level, which will be discussed next in the light of a bi-directional feedback between the gut and the brain.

### Effects on cognition and behavior linking diet and cognition via the microbiome−gut−brain axis

While the number of interventional studies focusing on cognitive and mental health outcomes after adopting plant-based diets overall is very limited (see Section I above), one underlying mechanism of how plant-based diets may affect mood could involve signaling pathways on the microbiome−gut−brain axis^116–119^. A recent 4-week intervention RCT showed that probiotic administration compared to placebo and no intervention modulated brain activity during emotional decision-making and emotional recognition tasks^117^. In chronic depression it has been proposed that immunoglobulin A and M antibodies are synthesized by the host in response to gut commensals and are linked to depressive symptoms^120^. Whether the identified gram-negative bacteria might also play a role in plant-based diets remains to be explored. A meta-analysis on five studies concluded that probiotics may mediate an alleviating effect on depression symptomatic^121^—however, sample sizes remained rather small (*n* < 100) and no long-term effects were tested (up to 8 weeks).

Currently, several studies aim to identify microbial profiles in relation to disease and how microbial data can be used on a multimodal way to improve functional resolution, e.g. characterizing microbial profiles of individuals suffering from type-1 diabetes^122^. Yet, evidence for specific effects of diet on cognitive functions and behavior through changes in the microbiome remains scarce. A recent study indicated the possibility that our food choices determine the quantity and quality of neurotransmitter-precursor levels that we ingest, which in turn might influence behavior, as shown by lower fairness during a money-redistribution task, called the ultimatum game, after a high-carbohydrate/protein ratio breakfast than after a low-ratio breakfast^123^. Strang et al. found that precursor forms of serotonin and dopamine, measured in blood serum, predicted behavior in this task, and precursor concentrations were dependent on the nutrient profile of the consumed meal before the task. Also on a cross-sectional level tryptophan metabolites from fecal samples have been associated with amygdala-reward network functional connectivity^124^. On top of the dietary composition per se, the microbiota largely contributes to neurotransmitter precursor concentrations; thus, in addition to measuring neurotransmitter precursors in the serum, metabolomics on fecal samples would be helpful to further understand the functional role of the gut microbiota in neurotransmitter biosynthesis and regulation^125^.

Indicating the relevance of gut microbiota for cognition, a first human study assessing cognitive tests and brain imaging could distinguish obese from nonobese individuals using a microbial profile^126^. The authors found a specific microbiotic profile, particularly defined by Actinobacteria phylum abundance, that was associated with microstructural properties in the hypothalamus and in the caudate nucleus. Further, a preclinical study tested whether probiotics could enhance cognitive function in healthy subjects, showing small effects on improved memory performance and reduced stress levels^127^.

A recent study could show that microbial composition influences cerebral amyloidogenesis in a mouse model for Alzheimer’s disease^128^. Health status of the donor mouse seemingly mattered: fecal transplants from transgenic mice had a larger impact on amyloid beta proliferation in the brain compared to wild-type feces. Translational interpretations to humans should be done with caution if at all—yet the results remain elucidative for showing a link between the gut microbiome and brain metabolism.

The evidence for effects of strictly plant-based diets on cognition is very limited. For other plant-based diets such as the Mediterranean diet or DASH diet, there are more available studies that indicate protective effects on cardiovascular and brain health in the aging population (reviewed in refs. ^129,130^). Several attempts have been made to clarify potential underlying mechanisms, for example using supplementary plant polyphenols, fish/fish-oil consumption or whole dietary pattern change in RCTs^131–137^, yet results are not always equivocal and large-scale intervention studies have yet to be completed.

The overall findings of this paragraph add to the evidence that microbial diversity may be associated with brain health, although underlying mechanisms and candidate signaling molecules remain unknown.

## Conclusion

Based on this systematic review of randomized clinical trials, there is an overall robust support for beneficial effects of a plant-based diet on metabolic measures in health and disease. However, the evidence for cognitive and mental effects of a plant-based diet is still inconclusive. Also, it is not clear whether putative effects are due to the diet per se, certain nutrients of the diet (or the avoidance of certain animal-based nutrients) or other factors associated with vegetarian/vegan diets. Evolving concepts argue that emotional distress and mental illnesses are linked to the role of microbiota in neurological function and can be potentially treated via microbial intervention strategies^19^. Moreover, it has been claimed that certain diseases, such as obesity, are caused by a specific microbial composition^138^, and that a balanced gut microbiome is related to healthy ageing^111^. In this light, it seems possible that a plant-based diet is able to influence brain function by still unclear underlying mechanisms of an altered microbial status and systemic metabolic alterations. However, to our knowledge there are no studies linking plant-based diets and cognitive abilities on a neural level, which are urgently needed, due to the hidden potential as a dietary therapeutic tool. Also, further studies are needed to disentangle motivational beliefs on a psychological level that lead to a change in diet from causal effects on the body and the brain mediated e.g., by metabolic alterations or a change in the gut microbiome.

## Supplementary information

Suppl. Table 1

Suppl. Figure 1
