# Supplementary material for: The effects of plant-based diets on the body and the brain: a systematic review
Source: Transl Psychiatry. 2019 Sep 12;9:226. doi: 10.1038/s41398-019-0552-0 (PMC6742661; doi:10.1038/s41398-019-0552-0)

**Suppl. Table 1: Characteristics of conventional, vegetarian and vegan diets.** International vegetarian and non-vegetarian marks as indicated by the Food Safety and Standards Authority of India (taken from https://en.wikipedia.org/wiki/Vegetarian_and_non-vegetarian_marks accessed on 25 Aug 2018). Information on macro- and micronutrient content has been synthesised on the basis of the reviewed literature.


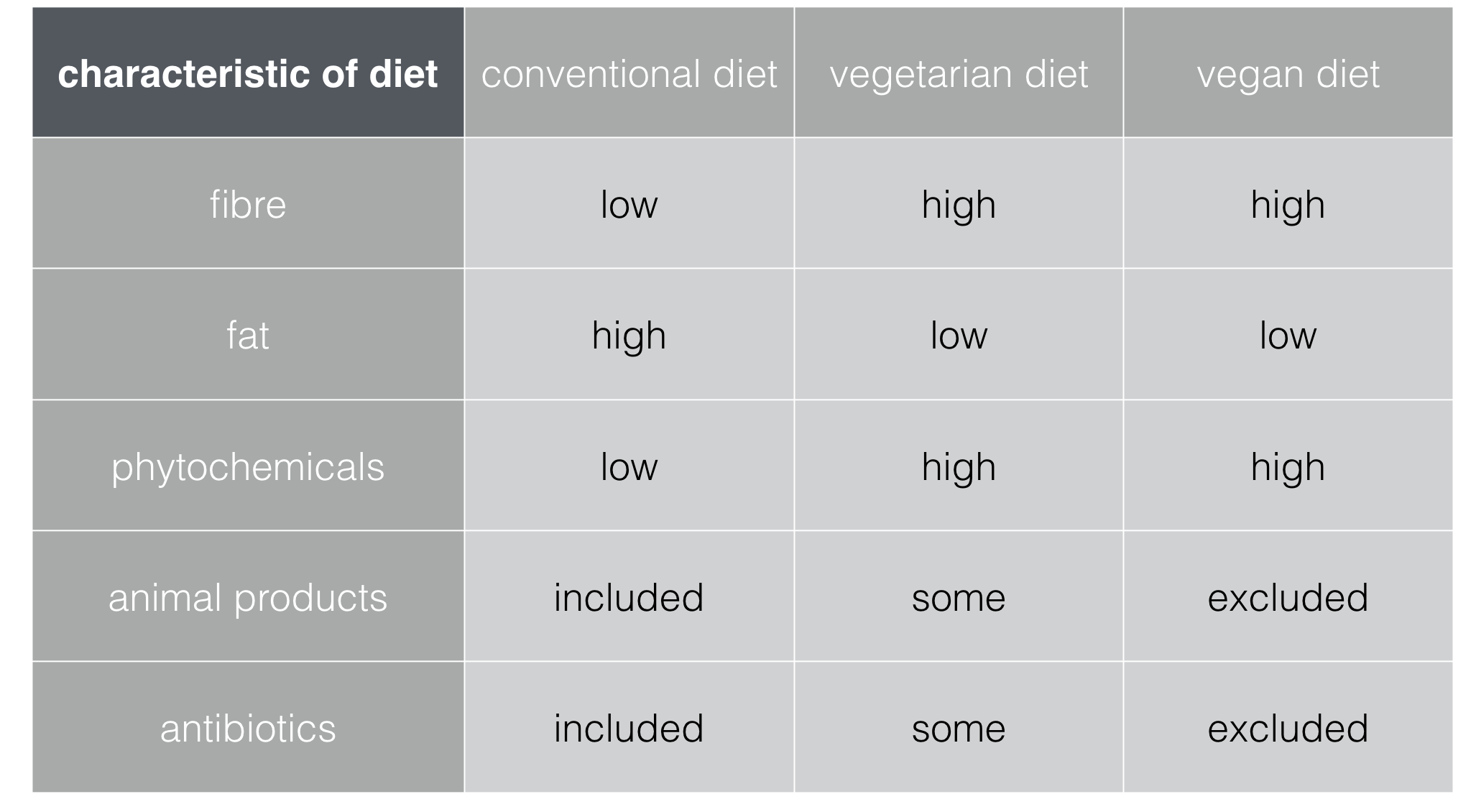

Supplement: Supplementary file 1 — Suppl. Table 1 [file 41398_2019_552_MOESM1_ESM.docx]
