# Supplementary figures and images for: The effects of plant-based diets on the body and the brain: a systematic review
Source: Transl Psychiatry. 2019 Sep 12;9:226. doi: 10.1038/s41398-019-0552-0 (PMC6742661; doi:10.1038/s41398-019-0552-0)

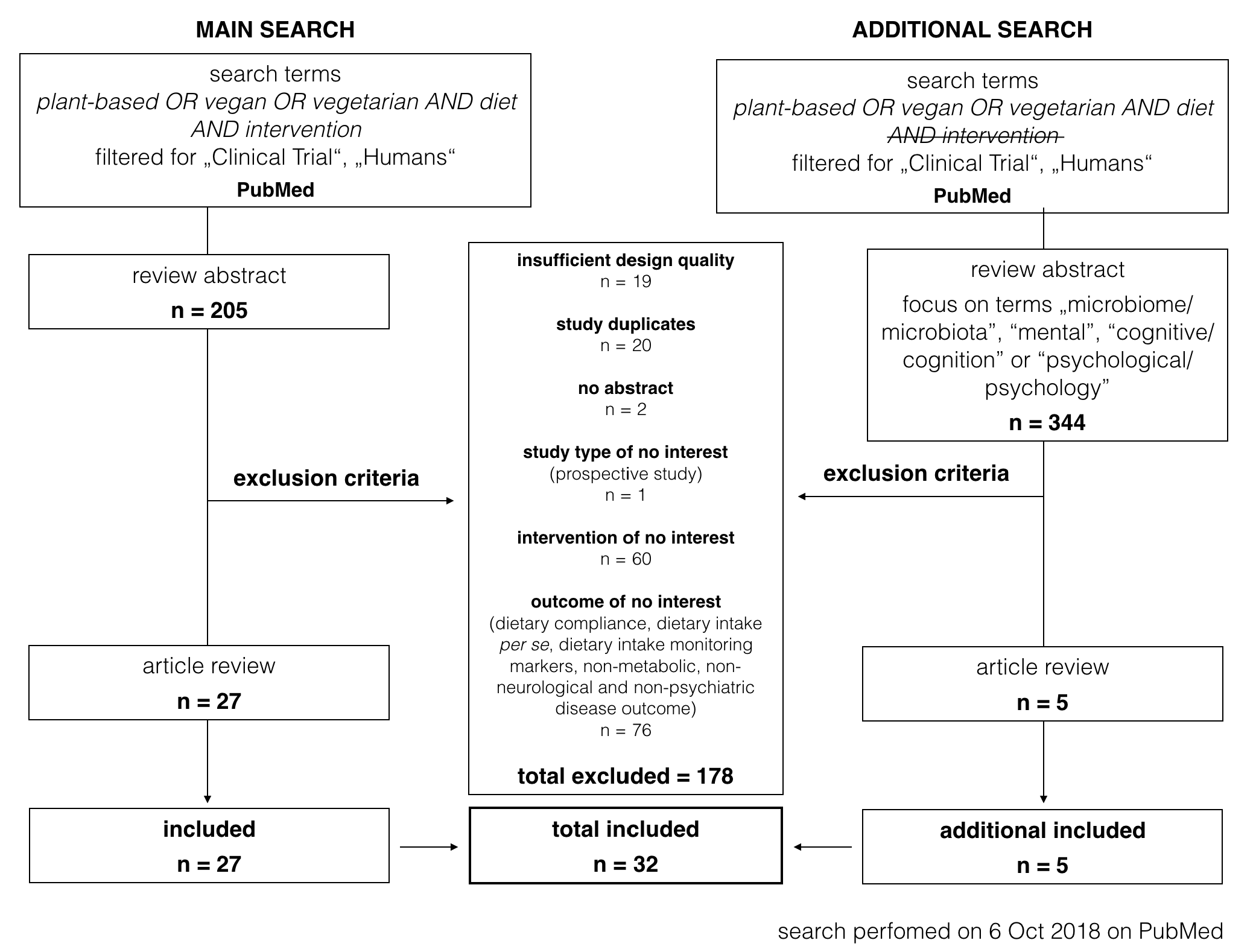


**Suppl. Figure 1: Flowchart of literature search** performed on 6 Oct 2018 on PubMed.

Supplement: Supplementary file 2 — Suppl. Figure 1 [file 41398_2019_552_MOESM2_ESM.docx]
